# A Narrative Review on the Role of AMPK on De Novo Lipogenesis in Non-Alcoholic Fatty Liver Disease: Evidence from Human Studies

**DOI:** 10.3390/cells10071822

**Published:** 2021-07-19

**Authors:** Christian von Loeffelholz, Sina M. Coldewey, Andreas L. Birkenfeld

**Affiliations:** 1 Department of Anesthesiology and Intensive Care Medicine, Jena University Hospital, 07747 Jena, Germany; Sina.Coldewey@med.uni-jena.de; 2 Septomics Research Center, Jena University Hospital, 07747 Jena, Germany; 3 Center for Sepsis Control and Care, Jena University Hospital, 07747 Jena, Germany; 4 Department of Diabetology Endocrinology and Nephrology, University Hospital Tübingen, Eberhard Karls University Tübingen, 72074 Tübingen, Germany; Andreas.Birkenfeld@med.uni-tuebingen.de; 5 Department of Therapy of Diabetes, Institute of Diabetes Research and Metabolic Diseases in the Helmholtz Center Munich, Eberhard Karls University Tübingen, 72074 Tübingen, Germany; 6 Division of Diabetes and Nutritional Sciences, Rayne Institute, King’s College London, London SE5 9RJ, UK

**Keywords:** lipotoxicity, insulin resistance, free fatty acids, steatohepatitis, type 2 diabetes, skeletal muscle, adipose tissue, diacylglycerol

## Abstract

5′AMP-activated protein kinase (AMPK) is known as metabolic sensor in mammalian cells that becomes activated by an increasing adenosine monophosphate (AMP)/adenosine triphosphate (ATP) ratio. The heterotrimeric AMPK protein comprises three subunits, each of which has multiple phosphorylation sites, playing an important role in the regulation of essential molecular pathways. By phosphorylation of downstream proteins and modulation of gene transcription AMPK functions as a master switch of energy homeostasis in tissues with high metabolic turnover, such as the liver, skeletal muscle, and adipose tissue. Regulation of AMPK under conditions of chronic caloric oversupply emerged as substantial research target to get deeper insight into the pathogenesis of non-alcoholic fatty liver disease (NAFLD). Evidence supporting the role of AMPK in NAFLD is mainly derived from preclinical cell culture and animal studies. Dysbalanced de novo lipogenesis has been identified as one of the key processes in NAFLD pathogenesis. Thus, the scope of this review is to provide an integrative overview of evidence, in particular from clinical studies and human samples, on the role of AMPK in the regulation of primarily de novo lipogenesis in human NAFLD.

## 1. Introduction

The healthy liver displays remarkable metabolic plasticity with hepatocytes in concert with a variety of other cell types, readily switching between various metabolic tasks. These transitions are regulated by numerous factors including hormones, nutrients, and further influences. However, with the onset of non-alcoholic fatty liver disease (NAFLD), this highly balanced physiological homeostasis becomes compromised [1].

It was shown in vivo that de novo lipogenesis (DNL) contributes substantially to liver fat storage in humans [2]. One hallmark finding in that matter has been the discovery of the regulation of lipid metabolism by 5′AMP-activated protein kinase (AMPK) via phosphorylation and inactivation of acetyl CoA carboxylase (ACC), the enzyme catalyzing the first and rate limiting step in DNL [3,4,5,6]. Moreover, a close association of ectopic lipid deposition with insulin resistance is well recognized. This supports the hypothesis of a crucial role of AMPK as molecular node between the epidemic pathologies obesity, type 2 diabetes (T2D), and NAFLD [7,8,9,10,11,12,13,14].

The following sections summarize available data on AMPK in the human fatty liver. Mechanisms governing the role of AMPK in liver fibrosis and inflammation will not be discussed in detail here (reviewed in [15,16]). Instead, we will focus on AMPK as a nexus between lipid metabolism and insulin resistance, with a specific scope on human studies researching DNL. Potential therapeutic benefits of AMPK activation will be discussed on the background of the spectrum of NAFLD pathogenesis.

## 2. The Epidemic of NAFLD

Current research suggests NAFLD as one of the most important causes of liver disease, probably emerging as the leading cause of end stage liver illness in future decades [17,18]. This epidemic in chronic liver dysfunction parallels the pandemic rise in obesity [19]. NAFLD is reported to affect 30% and more of the European population, with even higher or comparable incidences in the United States, Australia, India, and other countries [17,18]. NAFLD is described as a hepatic manifestation of metabolic syndrome and is nowadays considered a multisystem disease [1]. It is closely related to obesity, T2D, cardiovascular disease [20,21,22,23,24], associated with chronic renal pathologies [25,26,27], and with the incidence of hepatocellular carcinoma (HCC) and other malignancies [28,29,30,31]. Meanwhile, advanced NAFLD is the second leading and most rapidly growing etiology of HCC related liver transplantation in the USA [32]. After organ transplantation, NAFLD patients have an increased risk of premature death from cardiovascular complications and sepsis [33]. Moreover, liver transplant recipients due to advanced stages of NAFLD have a higher risk of post-transplant de novo T2D [34,35]. Our group has shown that NAFLD subjects undergoing major hepatic surgery frequently show impaired functional liver recovery [36]. In cardiac surgery, NAFLD is a predictor of high vasopressor support, of extrahepatic organ dysfunction, and of a longer intensive care and hospital stay [37].

**Figure 1:**
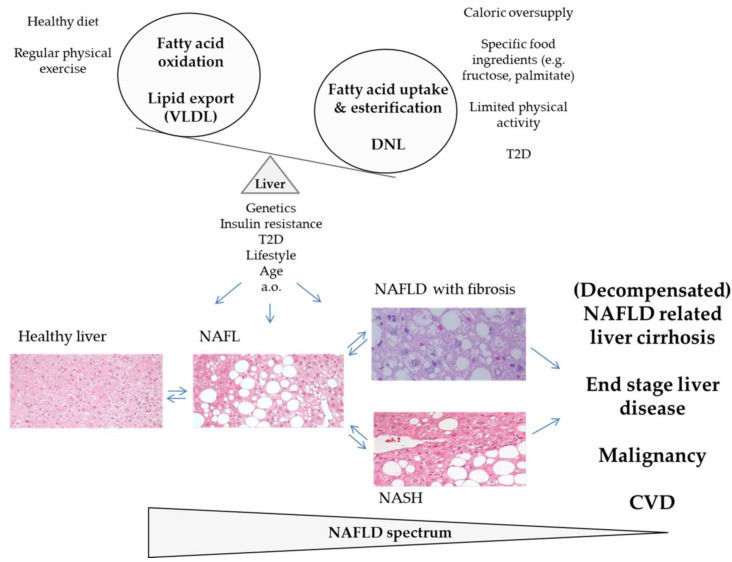
Spectrum of the NAFLD disease (adapted according to [28,50,51]). Human NAFLD develops when hepatic triacylglycerol accumulation (resulting from increased hepatic fatty acid uptake with esterification into triacylglycerol and from DNL) exceeds the rate of hepatic triacylglycerol catabolism (fatty acid oxidation and export as VLDL). NAFL can either progress to NASH and a variable percentage of NAFL patients develops signs of liver fibrosis independent from major liver inflammation. Both entities can result in end stage liver disease and clinical endpoints with poor prognosis quoad vitam. The factors triggering NAFLD progress are incompletely understood, particularly at the molecular level. Pathologies such as age, T2D and insulin resistance have been identified to play promoting role. CVD, cardiovascular disease; DNL, de novo lipogenesis; NAFLD, non-alcoholic fatty liver disease; NAFL, non-alcoholic fatty liver; NASH, non-alcoholic steatohepatitis; TAG, triacylglycerol; T2D, type 2 diabetes; VLDL, very-low-density lipoprotein.

The pathogenesis of NAFLD begins with triacylglycerol accumulation in the liver. NAFLD is defined as the presence of cytoplasmic lipid droplets in more than 5% of hepatocytes or triacylglycerols exceeding the 95th percentile for subjects with negative alcoholic, viral and autoimmune liver disease [38,39,40,41,42]. It was thought that the course of NAFLD would more or less intrinsically follow the so-called “two hit hypothesis” [43,44] based on the appearance of steatosis (first hit) and followed by a second hit leading to inflammation, hepatocyte injury, and fibrosis (second hit). This pathology is termed non-alcoholic steatohepatitis (NASH). Bland steatosis (non-alcoholic fatty liver, NAFL) was long believed to follow a relatively benign course [45]. It is now known that within relatively short time periods a subgroup of each, NASH and NAFL patients can progress from non-fibrotic stages to advanced liver fibrosis [28,46,47,48,49,50], potentially resulting in subsequent cirrhosis and organ failure [28,41,42]. Together, the course of human NAFLD can be highly variable and the current terminology “NAFLD” represents an umbrella term encompassing a broad spectrum of pathologic entities (see Figure 1), probably including “cryptogenic” liver fibrosis/cirrhosis [50,51,52]. NAFLD-related liver fibrosis/cirrhosis remain the clinical endpoints with the clearest evidence of a poor patient prognosis quoad vitam [28,29,49,50,51,52,53].

Apart from genetic aspects, various molecular influences are thought to be responsible for disease onset and progression. NAFLD is therefore increasingly termed metabolic associated fatty liver disease, MAFLD [54,55,56,57]. Of these, hepatocellular lipo-apoptosis and pyroptosis are considered key factors [58,59]. Fatty acid induced hepatocyte apoptosis is termed lipo-apoptosis, while pyroptosis follows activation of inflammatory caspases, leading to pore membrane formation, cell swelling, and massive release of pro-inflammatory contents. Interestingly, under each condition dysbalanced DNL and fat oxidation potentially play a role [57,58,59,60,61], pointing to the importance of AMPK as a master controller of both pathways.

## 3. Structure and Physiological Regulation of AMPK

Eukaryotic cells adapt to environmental energetic fluctuations by constantly managing energy consumption and the capacity to produce adenosine triphosphate (ATP). ATP is broken down into adenosine diphosphate (ADP) and can be further converted to adenosine monophosphate (AMP). When intracellular ATP levels decrease while AMP levels rise, cellular metabolism must shift towards ATP-producing catabolic pathways [62]. AMPK was recognized as mammalian homolog of a stress sensing kinase in yeast that is critical for cellular survival under conditions of marginal energy supply [5,63,64]). AMPK becomes allosterically activated by binding adenine nucleotides and thereby functions as a sensor of intracellular energy homeostasis [65,66].

**Figure 2:**
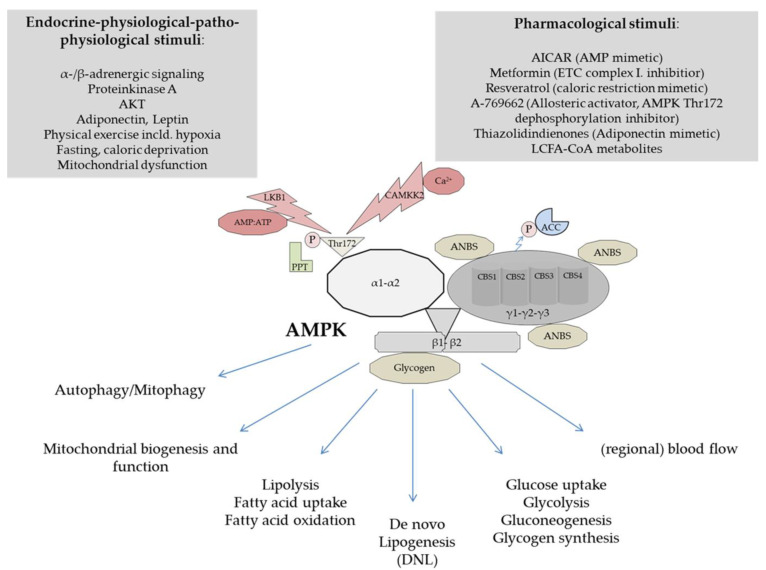
Schematic model of structure and overview of selected functional aspects of the AMPK heterotrimer (the figure was adapted according to ([70]). ACC, acetyl-CoA carboxylase; AICAR, 5-aminoimidazole-4-carboxamide-1-b-D-ribofuranoside; AMPK, AMP-activated protein kinase; ANBS, adenine nucleotide binding site; CaMKK2, Ca^2+^/calmodulin-dependent protein kinase kinase 2; CBM, carbohydrate binding module; CBS, cystathionine-β-synthase repeats; ETC, electron transport chain; LCFA-CoA, long chain fatty acid coenzyme A; LKB, liver kinase B1; PPT, protein phosphatase; Thr172, threonine 172. For further explanations please see main text.

From a biochemical perspective AMPK is a phylogenetically highly conserved heterotrimeric serine/threonine protein kinase complex consisting of one catalytic (α) and two regulatory subunits (β, γ). Various isoforms of each subunit are encoded by individual genes [65,67,68,69]. As shown in Figure 2 two α-(α1, α2), two β-(β1, β2), and three γ-subunits (γ1, γ2, γ3) can form the AMPK complex [70]. They are expressed tissue specific and the resulting AMPK subunits can either exert overlapping or distinct functions [70,71,72,73,74,75,76,77]. Depending on isoform expression and combination, twelve heterotrimeric holoenzyme-variants can theoretically be deduced [69]. Excellent reviews on structure, tissue related subunit expression, the multitude of phosphorylation sites, and subcellular localization are available, to which the specifically interested reader is referred [65,69,77,78,79,80].

Multiple endocrine, (patho-) physiological, and pharmacological stimuli are known to modulate AMPK (see Figure 2). The phosphorylation of a conserved threonine-172 (Thr172) residue in the activation loop of the N-terminal region of the α-subunit is known to be required for full AMPK activation by upstream kinases in mammalian cells (reviewed in [77]). Using purified recombinant enzyme preparations, Suter et al. have shown that upstream kinase activation in the presence of saturating AMP effects resulted in a 1000-fold increased activation of α1-β1-γ1- and α2-β2-γ1 AMPK heterotrimers [81]. Interestingly, AMP did not enforce kinase dependent phosphorylation, at least under the research conditions chosen by the authors, but protein phosphatase mediated deactivation by site specific dephosphorylation was prevented [81]. Meanwhile, these findings have been challenged and complemented by a broad body of further research, and AMP binding is suggested to modify the AMPK protein by allowing more effective upstream stimulation. Moreover, augmented allosteric activation of still Thr172 phosphorylated AMPK by AMP and further mechanisms have been discussed [67,82,83,84]. Together, available evidence generally supports the view that for full enzyme activation adenine nucleotide (AMP, ADP) binding is essential for Thr172 phosphorylation in the activation loop of the α-catalytic domain [65,83]. Since the scope of this review is on the metabolic function of AMPK in the liver, it should be further noted that quantitative analyses revealed that both the α1- and α2-subunit are expressed in hepatocytes [78]. Adenine nucleotide binding is realized by three adenine nucleotide binding sites (ANBS), which are localized in three out of four cystathionine-β-synthase repeats (CBS1-4) in the AMPK γ-subunit [77]. CBS4 was shown to exclusively and permanently bind AMP [85,86], while CBS1 and CBS3 competitively bind AMP, ADP, or ATP, depending on their respective cellular abundance [65]. This elegantly enables the AMPK protein complex to rapidly sense changes in AMP:ATP ratio and immediately balance cellular energy supply [82]. Another structural key element in the AMPK γ-subunit is CBS2. CBS2 is discussed to be closely related to ACC phosphorylation [87]. The β-subunit is of extraordinary importance for mediating metabolic effects and contrasting with humans, the rodent liver predominately expresses β1, while the β2-subunit appears to be of minor importance in this species [67,71,88,89,90,91]. The β-subunit enables AMPK to bind glycogen via a carbohydrate binding module (β-CBM), augmenting its energy sensitizing properties [82]. Moreover, it was actually shown by Steinberg’s group that activated long chain fatty acid esters can allosterically activate β1-subunit containing isoforms and thereby increase lipid oxidation through phosphorylation of ACC [92].

Two upstream kinases are responsible for AMPK activation (see Figure 1). The tumor suppressor liver kinase B1 (LKB1) is constitutively active and considered to be the predominant upstream kinase [93]. Adenine nucleotide independent activation of AMPK activity in response to hormonally stimulated changes in cytosolic calcium concentrations is mediated by the Ca^2+^/calmodulin-dependent protein kinase kinase 2 (CaMKK2) [94,95,96]. A role in AMPK activation, still not well characterized, was also discussed for the TGFβ activated kinase-1 (TAK1) (reviewed in [77]). Specific protein phosphatases (PP2A and 2C) antagonize upstream kinase signaling and inhibit AMPK activity by dephosphorylation of Thr172.

AMPK activation results in immediate posttranslational modification of regulatory downstream proteins involved in glycolysis, lipid oxidation, and further cellular processes engaged in short term inducible nutrient combustion. Long term regulatory effects are controlled by phosphorylation of transcription factors and co-activators that regulate each of these metabolic functions [65,66]. In addition to reducing AMP:ATP ratio activation of AMPK affects whole body fuel utilization by inducing lipolysis and fatty acid oxidation [97,98]. Moreover, growing evidence points to regulation of mitochondrial homeostasis by stimulation of biogenesis and functional quality via controlling autophagy/mitophagy (reviewed in [99,100]). Effects on vasculature as mediated by activation of endothelial NO synthase (eNOS) with influence on blood flow and thereby cellular nutrient supply were reported (reviewed in [101]). Apart from metabolic functions it should be noted that a role of AMPK has also been suggested for carcinogenesis (reviewed in [76,80]).

Due to the core functional position in metabolism, AMPK was recognized as a promising pharmacologic target structure. Metfomin, a drug widely used in the therapy of T2D, exerts some of its main effects by AMPK activation in the liver and skeletal muscle [102,103]. Increasing AMPK activity is widely viewed as viable therapeutic strategy [15]. Stimulation of AMPK not alone inhibits ACC1, but further leads to concomitant phosphorylation of ACC2 and activation of malonyl CoA decarboxylase (MCD), resulting in an acute reduction of cytosolic malonyl CoA [104]. Malonyl CoA is well known as negative regulator of fatty acid oxidation by virtue of its ability to inhibit carnitine palmitoyl-transferase 1 (CPT1). Thus, when malonyl CoA levels decrease, CPT1 remains uninhibited and fatty acid oxidation becomes accelerated [105,106,107,108].

This unique effect in lipid metabolism excellently symbolizes the central position of AMPK in balancing fuel homeostasis. The latter, however, becomes substantially impaired under conditions of chronic caloric oversupply, potentially resulting in ectopic lipid deposition and insulin resistance [109].

## 4. Interactions of Fatty Acids with Insulin Signaling: A Lipotoxic Event from a Human Perspective

One cellular dysfunction that can exaggerate DNL and by itself becomes promoted by the resulting rise in harmful lipid species is insulin resistance [14,56,58,61,110,111,112,113,114,115]. Insulin resistance mainly affects three metabolic key organs: the liver, skeletal muscle, and adipose tissue. Peripheral insulin resistance is defined by impaired suppressibility of lipolysis and reduced glucose transporter 4 (GLUT4) mediated glucose uptake in adipose tissue, and by reduced GLUT4 dependent glucose uptake with decreased glycogen synthesis and impaired anabolic/anticatabolic response to insulin in skeletal muscle. Hepatic insulin resistance is defined by decreased capacity to synthesize glycogen and impaired suppression of glycogenolysis, gluconeogenesis, and DNL in the liver after insulin stimulation [109,113,116,117,118].

**Figure 3:**
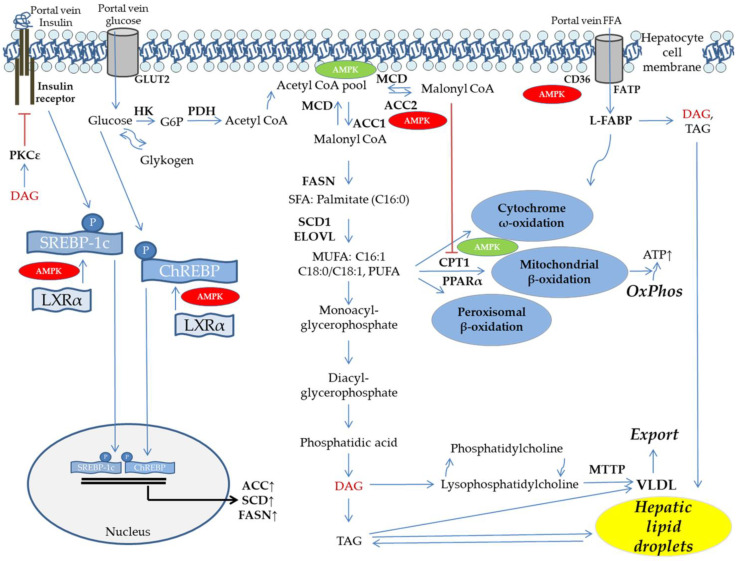
Lipid metabolism in the human liver. Schematic presentation of selected AMPK related pathways and signaling mechanisms relevant in human NAFLD. Hepatic DNL and FFA uptake can contribute to liver lipid accumulation, while fatty acid oxidation and lipid export as VLDL particles reduce hepatic TAG. Effects of AMPK known from experimental and animal models are indicated, inhibitory effects of AMPK are colored in red, promoting effects are shed in green (adapted from and according to [16,76]). Inhibitory signaling and DAG are colored brownish. ACC, acetyl CoA carboxylase; AMPK, AMP-activated protein kinase; ATP, adenosine triphos-phate; CD, cluster of differentiation; ChREBP carbohydrate regulatory element binding protein; CPT, carnitine palmitoyl-transferase; DAG, diacylglycerol; ELOVL, elongation of very long chain fatty acid; FASN, fatty acid synthase; FATP, fatty acid transport protein; FFA, free fatty acids; GLUT, glucose transporter; HK, hexokinase; L-FABP, liver fatty acid binding protein; MCD, mal-onyl CoA decarboxylase; MTTP, microsomal triglyceride transfer protein; MUFA, monounsaturated fatty acids; OxPhos, oxidative phosphorylation; PDH, pyruvate dehydrogenase; PPAR, PPAR, peroxisome proliferator activated receptor; PUFA, polyunsaturated fatty acids; SCD, stearoyl CoA desaturase; SFA, saturated fatty acids; SREBP, sterol regulatory element binding protein; TAG, triacylglycerol; VLDL, very low density lipoprotein.

A complete accounting for all molecular pathways involved in the pathogenesis of insulin resistance is beyond the scope of this review (reviewed in [12,109,118]. However, Figure 3 provides an overview of selected aspects related to the potential role of AMPK. Of the lipotoxic intermediates responsible for impaired insulin signaling, the DNL and fatty acid esterification product diacylglycerol represents an extraordinary candidate [14,109,118]. Diacylglycerol activates atypical protein kinase C (PKC)ε and θ, resulting in acutely impaired insulin signaling along with further candidates, such as PKCδ [9,12,109,116,119,120,121,122]. This excellently embodies the lipotoxic potential of a chronically exaggerated DNL. At the same time obesity, NAFLD and T2D become integrated in a pathophysiological model. This model supports the view of AMPK as a potential “upstream key player” in the transition from overnutrition to NAFLD and insulin resistance. For a better understanding of the potential role of AMPK in this setting it is, however, inevitable to get a deeper understanding of the close interconnections between insulin resistance and ectopic lipids.

Ectopic lipid deposition is closely associated with insulin resistance under human in vivo conditions [7,13,61,123]). In this regard, the group of Bril et al. recently challenged the current cut-off of intrahepatic triacylglycerol content of ~5% because of its insufficiently validated association with metabolic and histological outcome [124]. They included 352 subjects in a cross-sectional study and characterized this cohort by using gold standard methods. It was shown that hepatic insulin sensitivity was reduced when triacylglycerol levels approached ~2% of liver tissue and remained impaired, regardless of further lipid accumulation. The capacity of glucose uptake in skeletal muscle was gradually decreased under conditions of low liver fat, but remained stable after hepatic triacylglycerol levels had reached ~6%. Adipose tissue (AT) insulin sensitivity showed a continuous worsening across the full spectrum of hepatic fat accumulation, suggesting a liver-periphery cross-talk. Unexpectedly histological severity of fatty liver disease (e.g., cell death and inflammation) was of minor importance [124]. Thus, under human in vivo conditions the degree of hepatic lipid accumulation is a direct barometer of whole body insulin sensitivity, or reciprocally insulin resistance [8,11,125]. More evidence to support this comes from numerous human intervention studies. It was shown that significant body weight reduction resulted in up to ~81% liver fat reduction and normalization of insulin resistance [126,127,128,129]. Our group has evidenced a role for the human liver fatty acid binding protein (L-FABP) [130]. L-FABP is a highly conserved factor of hepatocellular fatty acid uptake (see Figure 3). Studies in knockout models suggest that specific amino acid replacements are associated with functional impairment [131,132]. We exposed healthy carriers of a common amino acid replacement (Ala/Ala94) vs. age-, sex-, and BMI-matched wild-type (Thr/Thr94) controls to a lipid infusion protocol, aiming at plasma free fatty acid (FFA) levels typically observed in T2D. With the lipid challenge an induction of insulin resistance and rise in plasma glucose was detectable. Intriguingly, a significant genotype vs. lipid-treatment interaction with reduced glycogenolysis and less severe hyperglycemia was observed in Ala/Ala94 carriers [130], suggesting less impaired insulin signaling due to reduced hepatocellular uptake of lipotoxic FFA species. The latter finding was specified by work of Clore et al., who by application of palm oil evidenced a lipid species dependent effect [133]. Palm oil provides high concentrations of palmitic acid/palmitate, exerting unfavorable effects including AMPK inhibition [134]. Remarkably, palmitic acid/palmitate is an end product of DNL (see Figure 3) and at the same time an important precursor of diacylglycerol [16].

Together, human in vivo research suggests lipotoxic effects of DNL derived lipid species. The fact that caloric restriction is capable of reversing these effects raises the hypothesis that AMPK represents a potential molecular target in NAFLD treatment.

## 5. DNL and FFA Flux Determine Hepatic Lipid Dysbalance

It was shown that in starved obese NAFLD patients ~25% of the FFA entering the liver are taken up, mainly via supply from subcutaneous adipose tissue (SCAT) ([2]; reviewed in [61]). Therefore, AT insulin resistance is discussed as key driver of excess hepatic lipid storage. After a meal circulating FFA account for ~60% and DNL for 25–30% of excess liver triacylglycerol storage, while dietary lipids range as negligible contributors ([2,135], reviewed in [61]). Complementary human studies support these results [136,137,138]. DNL is 3-fold higher in NAFLD subjects compared to healthy controls [136]. NAFLD patients failed to suppress DNL under starving conditions since it took 16.5 h without food intake to suppress DNL to basal levels [136]. Fasting hyperinsulinemia is a driver of overflowing DNL [139]. The NAFLD patients studied by Lambert et al. showed elevated insulin levels, but were matched for main demographic and metabolic confounders [136]. The latter is supported by a further study showing that in hyperinsulinemic patients undergoing a high carbohydrate diet, fasting DNL increased up to 5-fold [140].

Glucose is the primary substrate of hepatic DNL [62] and diets rich in carbohydrate, but rather low in dietary lipids, are long recognized to be capable of exaggerating DNL. This has particularly been shown in the presence of overweight and hyperinsulinemia [141,142,143,144,145,146]. Under slightly hypercaloric conditions, a carbohydrate rich diet can contribute 211 g of newly synthesized fat within a 21 day period [147]. With massive carbohydrate overfeeding, after saturation of glycogen stores and with a continuously positive energy balance, exaggerated hepatic DNL may result in up to 150 g of newly synthesized fat per 24 h in humans [148]. Moreover, fructose when consumed in substantial amounts (sugared beverages) can promote DNL independently from hyperinsulinemia [146]. These data mark DNL as one predominant fueling process of human in vivo NAFLD under conditions of a “Western diet”. It should be further noted that the degree of liver steatosis in the NAFLD subjects studied by Donelly et al. ranged about 38%, while it was ~18% in the patients investigated by Lambert et al. [2,136]. This could indicate that hyperinsulinemia induced DNL is of more significance in early stages of NAFLD pathogenesis, although this remains to be substantiated.

## 6. Mitochondrial Function, Fat Oxidation, and Lipid Export

Under physiological conditions the accrual of fatty acids due to uptake from the plasma and synthesis from DNL is balanced by oxidation and by plasma secretion as VLDL triglycerides. FFA become mainly oxidized by mitochondrial β-oxidation in muscle, the liver, and further organs, and by peroxisomal β-oxidation and microsomal ω-oxidation in the liver [16]. With onset of insulin resistance, oxidation and secretion, are insufficient to compensate for excess lipid accumulation (see Figure 1).

No direct measurement of mitochondrial oxidation capacity is available under human in vivo conditions. Therefore, various indirect methods are used to estimate mitochondrial function (i.e., plasma β-hydroxybutyrate, magnetic resonance spectroscopy techniques). According to available evidence in humans it appears likely that in NASH mitochondrial function becomes significantly compromised and oxidation capacity decreases when compared to bland steatosis or healthy conditions [149,150,151,152,153,154,155]. Contradictory results are published for patients suffering from less severe NAFLD. Regarding mitochondrial oxidative capacity, available data either indicates a reduction [155], an unaltered function [156], or an increase [151,154,157]. These results should, however, be interpreted with caution, keeping the variable course of human disease in mind (see Figure 1). Subjects in the study of Petersen et al. had ~8% liver fat in contrast to the subjects of Sunny et al. (~17% liver fat) or Roden et al. (~27% liver fat). Thus, different patient groups in terms of NAFLD were likely studied [154,156,157], although liver histology was mostly not available [156,157]. A standardized differentiation of NAFL with or without fibrosis and NASH was therefore not possible, potentially introducing bias in the interpretation of the data.

FFA oxidation is estimated by indirect methods and evidence suggests an increase or decrease in human NAFLD [137,157,158], although none of these studies provided liver histology either. This is of relevance as in very advanced NAFLD (“cryptogenic”/“burnout” liver cirrhosis) steatosis can be reduced or become resolved [52]. Interestingly, administration of a lipid infusion protocol in patients with histologically proven NASH showed increased FFA oxidation capacity, at least within the 240 min time interval after onset of the challenge [159]. This evolves the hypothesis that fat oxidation could be differentially regulated under conditions of starvation and postprandially. However, methods of the latter study were limited and further research is needed [159]. Although not studied yet, it appears plausible that AMPK, due to its key position in mitochondrial β-oxidation, could be a significant hub in such settings.

Very long-, branched chain-, and unsaturated FFA primarily undergo oxidation in hepatic peroxisomes and microsomes (see Figure 3). Few data are available on this in NAFLD. We are aware of no human NAFLD study evaluating peroxisomal β-oxidation and only one addressing ω-oxidation, using 13C-methacetin-non-invasive stable isotope breath tests [151]. This study indicates significantly disturbed microsomal function in human NASH [151].

VLDL triglyceride mediated lipid export appears not to be a majorly inducible factor in terms of hepatocellular lipid overload (reviewed in [160]). Secretion of triacylglycerol enriched lipoproteins by the liver is increased in NAFLD, which can be clinically observed as hypertriglyceridemia. By ~6%, liver lipid content hypertriglyceridemia in humans becomes fully established [124]. At the same time, mainly triacylglycerols from exogenous lipids rather than from increased DNL become incorporated (reviewed in [160]).

Together, available evidence supports the view that to overcome dysbalanced lipid homeostasis, compensatory mechanisms are activated. In early disease, increased mitochondrial function and lipid oxidation capacity can be expected. Activation may vary on an individual basis and on NAFLD stage, finally resulting in functional decrease with advanced liver fibrosis. Excess lipolysis, hepatocellular FFA uptake, and increased (fasting) DNL due to insulin resistance are predominant factors to be considered, when aiming at preventing the disease onset and progression by means of AMPK activation.

## 7. The Role of AMPK in Human Fat Depots

Based on the close association of NAFLD and obesity, AT expansion is common in fatty liver patients. The shift of fat storage away from subcutaneous (SCAT) towards visceral white adipose tissue (VAT) is considered a predictor of increased risk profile [137,161].

**Figure 4:**
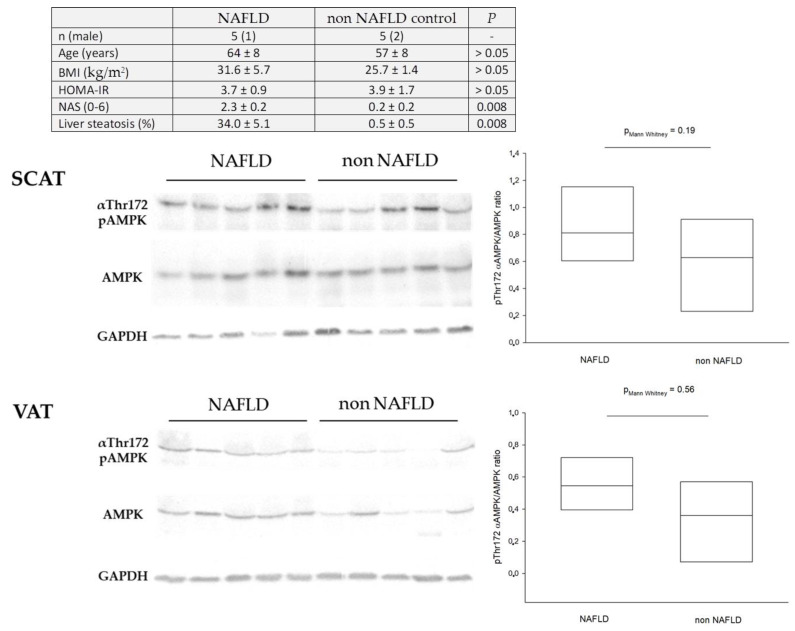
AMPK in human adipose tissue of NAFLD subjects. AMPK activity as assessed by the quotient of Thr172 phosphorylated to total AMPK was not significantly different in VAT and SCAT of NAFLD and non NAFLD subjects (*p* > 0.05). This corresponded to αAMPK mRNA expression in both AT depots (data not shown, *p* = 0.55 for SCAT and *p* = 0.84 for VAT). Subjects were matched for main confounders, i.e., age and insulin resistance, with comparable gender distribution between groups. NAFLD patients were obese, while non NAFLD controls were categorized to be overweight. AT was sampled during open abdominal surgery after an overnight starvation period in a human study researching the role of AMPK in the liver. AMPK, AMP-activated protein kinase; HOMA-IR, homeostasis model of insulin resistance; NAFLD, non-alcoholic fatty liver disease; NAS, NAFLD activity scoring; SCAT, subcutaneous adipose tissue; VAT, visceral adipose tissue.

AMPK is known to be expressed in AT and to exert relevant metabolic functions (reviewed in [15]). Under physiological conditions AMPK is primarily discussed to contribute to AT insulin sensitivity [15]. Expression appears to be higher in human SCAT compared to VAT, at least in morbid obesity (BMI ≥ 40 kg/m^2^; *n* = 17), and after short term starvation [162]. Reduced AMPK activity, as estimated by Thr172 phosphorylation of αAMPK in relation to total protein, positively correlated with insulin resistance in obese humans [163,164]. However, the number of studied subjects was limited, all were morbidly obese, and the homeostasis model of insulin resistance (HOMA-IR) as a relatively rough estimate was used to evaluate the efficacy of insulin signaling [163,164]. Our group has studied AMPK expression and activation in VAT and SCAT of NAFLD patients (see Figure 4). However, we were unable to observe any significant differences between groups, yet subjects were matched for main confounding factors. Contrasting with the studies of Ruderman’s group [163,164], our patients were not morbidly obese, and NAFLD was evaluated by gold standard methods. However, we did also use the HOMA-IR for estimating insulin resistance and did not perform functional analyses. Moreover, our control subjects were overweight and we cannot exclude that this could have biased the results. Grisouard et al. differentiated human preadipocytes in vitro and studied the effects of metformin stimulation on glucose uptake [165]. Metformin increased glucose uptake more than twofold, accompanied by a rise in GLUT4 and glucose oxidation. Silencing of AMPKα1 counteracted the effects [165]. Available evidence therefore supports the hypothesis that AMPK contributes to peripheral insulin sensitivity. Pharmacological AMPK activation in a manner supporting cellular glucose uptake with consecutively enforced FFA esterification could help to control for hyperlipolysis. Insulin mediated lipolysis suppression showed a continuous worsening with rising liver fat accumulation [124] and hyperlipolysis is considered a major contributor of NAFLD (reviewed in [61,118]). Hyperlipolysis is further aggravated when adipocytes under conditions of a repetitively positive energy balance become hypertrophic. This can result in systemic low-grade inflammation, as promoted by macrophages residing in AT with worsening of insulin resistance [14,61,166,167,168,169]. However, according to numerous experimental and animal models, the role of AMPK on AT lipolysis remains inconclusive, with existing evidence supporting both enhanced and suppressed lipolysis by AMPK [77,98,170,171,172,173,174,175,176]. We are aware of no research data specifically addressing this issue in human NAFLD. However, indirect evidence to support the hypothesis of suppressed lipolysis (and/or elevated fat oxidation) in humans via AMPK comes from a study using an AICAR stimulation protocol [97]. Given the percentage of AT in relation to total body mass, further research on this principle could provide interesting insights on the role of AMPK, particularly regarding peripheral insulin resistance in various AT depots [177]. This principle is also supported by animal data showing that abolishing AMPK in AT results in decreased whole body insulin sensitivity and enhanced liver triacylglycerol accumulation [178]. Furthermore, human adipocytes in principal have the capability of DNL [144]. However, in contrast to rodents it is known that AT is not a significant site of DNL in humans [179,180].

Few data are available on AMPK expression and activity in human adipose tissue. Results are mainly inconclusive and have to be interpreted as hypothesis-generating. Otherwise, animal and experimental data suggest a role for AMPK in AT.

## 8. Skeletal Muscle Is a Predominant Target Tissue of Insulin and Sensitive to AMPK Activation

Skeletal muscle is the most important site of insulin stimulated glucose disposal in human body (reviewed in [118,181]). Liver triacylglycerol content can be interpreted as direct barometer of whole-body insulin resistance, with maximally impaired insulin signaling in skeletal muscle being reached when hepatic triacylglycerol levels range about 6% [124]. AMPK is expressed at high levels in skeletal muscle, and under physiological conditions the kinase contributes to insulin sensitivity and insulin independent exercise related peripheral glucose uptake.

A number of studies have addressed the role of AMPK in human skeletal muscle. The majority of trials were of short duration and included limited subject numbers. Only one study considered NAFLD [182]. The studies can be categorized in (i) descriptive studies focusing basal AMPK activity in obesity/insulin resistance [183,184,185], (ii) interventional studies using AMPK mimetics under in vivo or ex vivo conditions [97,182,184,186,187,188], and (iii) exercise studies (e.g., [187,189,190,191,192]). It was shown that after 12–18 h of starvation basal AMPK activity and expression remained comparable in muscle of lean and obese subjects [184]. This was also reported for T2D patients and controls [183,185,186]. However, patients in several of these studies were under antidiabetic medication including metformin, with no relevant drug free interval before intervention [186,188]. Thus, the results must be interpreted carefully.

Steinberg et al., by using AICAR ex vivo stimulation, showed increased muscle α2AMPK activity with complementary downstream ACC phosphorylation and palmitate oxidation to a comparable extent in lean and obese subjects [184]. In T2D, skeletal muscle exposure to AICAR was capable of restoring glucose transport and cell surface GLUT4 to levels observed in healthy controls [186], and of decreasing plasma FFA and endogenous glucose production, while increasing glucose uptake [97]. Together, the majority of studies using AICAR resulted in short term activation of muscle AMPK along with clinical improvements. Comparable findings on AMPK are reported for exercise. With onset of physical activity, AMPK becomes acutely activated in skeletal muscle, particularly at low muscle glycogen concentrations, and mediates important adaptions of muscle tissue plasticity to exercise [190,191,192,193,194]. The predominant effect is the elevation of whole body insulin sensitivity [192]. This has also been proven in T2D [187,189]. Therefore, current evidence supports the view of significant muscle AMPK stimulation by exercise, and of an association with increased insulin sensitivity, which is of clinical relevance in NAFLD treatment [195]. By contrast, long term effects of “exercise mimetics” on AMPK in skeletal muscle are largely unknown. One prospective randomized double-blind placebo controlled cross-over study addressed the “caloric restriction mimetic” resveratrol in obese individuals [182]. Increased phosphorylation of AMPK in skeletal muscle, along with numerous effects on molecular downstream targets and some clinical parameters, were observed over a 30 day period. The reduction in hepatic triacylglycerol could be potentially attributed to a redistribution of ectopic lipids away from the liver and towards skeletal muscle. In terms of insulin resistance this resulted in small, albeit significant, effects on HOMA-IR [182]. The study principally supports the concept of longer-term AMPK activation in skeletal muscle as a promising pharmacological target structure. Otherwise, HOMA-IR was 2.80 ± 0.20 with placebo vs. 2.43 ± 0.24 (*p* = 0.03) under resveratrol, pointing to the question of the clinical relevance of such an effect. The effects on liver lipids were of comparable size. Thus, further studies are needed to confirm the results of this limited single center trial.

## 9. Dysregulation of Hepatic AMPK in Humans

DNL is a main contributor to hepatic triacylglycerol accumulation under conditions of caloric oversupply, with glucose representing the main DNL substrate. The quantitatively only relevant site of DNL in human metabolism is the liver [179]. Therefore, attempts to modulate DNL in a therapeutic manner by means of AMPK activation should primarily address hepatic isoforms. Under physiological conditions AMPK is considered to balance hepatocellular energy demands, with major influence on liver insulin sensitivity and lipid homeostasis [76].

**Figure 5:**
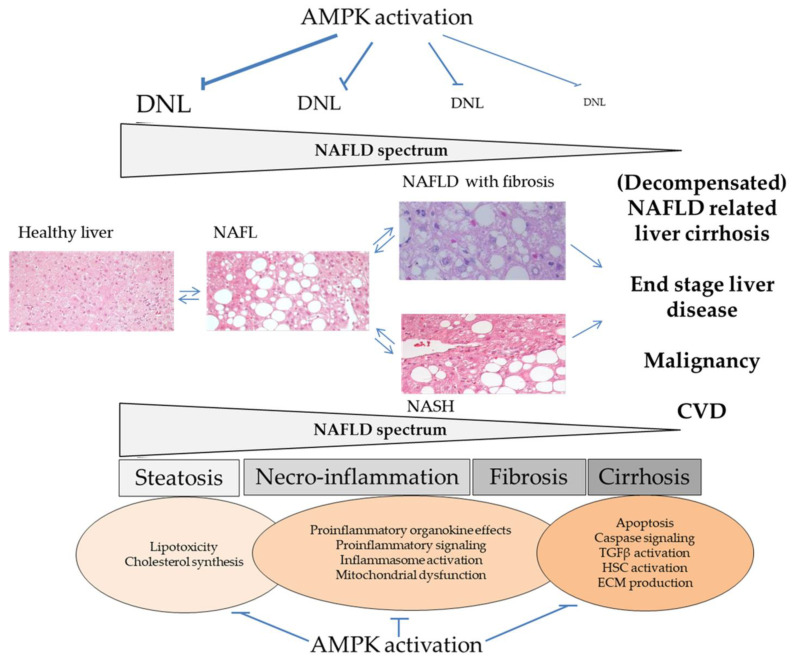
The potential role of AMPK activation along the human NAFLD spectrum. Upper part: DNL is under key control of AMPK due to inhibitory phosphorylation of ACC. Inhibition of DNL with related lipotoxicity could exert beneficial effect, although effects could become less significant with advancing disease. Lower part: Research of recent years has shown multiple effects with potentially beneficial impact on various stages of the NAFLD disease. The role of AMPK activation on DNL and lipotoxicity discussed in this review represents only one strategy of AMPK activation in the field of NAFLD (adapted from [203]). AMPK, AMP-activated protein kinase; ECM, extracellular matrix; HSC, hepatic stellate cells; NAFLD, non-alcoholic fatty liver disease; NAFL, non-alcoholic fatty liver; NASH, non-alcoholic steatohepatitis; TGF, transforming growth factor.

The main predictor of NAFLD is hepatic insulin resistance, defined by the failure of insulin to suppress endogenous glucose production, e.g., gluconeogenesis [61,196]. Under physiological conditions DNL, like gluconeogenesis, is under slow but potent transcriptional control by an insulin dependent mechanism [118]. When hepatocellular insulin signaling becomes attenuated DNL activation remains paradoxically uncompromised. This is referred to as selective insulin resistance, resulting in fasting hyperglycemia and uncontrolled DNL at the same time [197]. Corresponding to the human study of Lambert et al. [136], fasting hyperglycemia is a significant driver of DNL since glucose independently from insulin can enter hepatocytes in large amounts via GLUT2, and thereby enter glycolysis (see Figure 3). Dysregulation of glycolysis under circumstances of insulin resistance is complex (reviewed in [118]). The enzymes of the glycolytic pathway can be considered as an extended part of DNL, since the major function of glycolysis in hepatocytes is not to provide ATP, but to allow the transformation of carbohydrates into lipids [179]. The oxidative branch of the pentose phosphate pathway becomes activated in parallel, generating NADPH necessary for DNL [179]. With selective insulin resistance, DNL is therefore majorly influenced by combined hyperglycemia and hyperinsulinemia. As shown in Figure 3, this is mediated by transcription factors such as ChREBP (hyperglycemia) and SREBP-1c (hyperinsulinemia) on the molecular level. Otherwise, hepatic AMPK is considered to control for this situation under physiological conditions [16,77]. Yet, evidence from a broad spectrum of experimental and animal studies suggest hepatic AMPK dysfunction in NAFLD (reviewed in [15]). By contrast, only a few studies have evaluated AMPK in human liver. Kohjima et al. examined liver samples from *n* = 10 control subjects and *n* = 26 patients with histologically proven NAFLD [198,199]. They found significantly increased hepatic ACC1 mRNA expression, while FASN and SREBP-1c were not significantly elevated. Otherwise, several key enzymes of the mitochondrial and peroxisomal fatty acid oxidation machinery, along with some protective proteins of the cellular antioxidative system were upregulated. Interestingly, hepatic AMPK mRNA expression remained comparable between NAFLD and control subjects [198]. Our group evaluated *n* = 32 histopathologically proven NAFLD patients and included *n* = 16 non NAFLD control subjects [200]. Patients were mainly suffering from NAFL. Along with a liver lipidomic pattern characteristic for chronically activated DNL, we observed a marked upregulation of DNL key enzymes and related transcription factors in subjects suffering from severe liver steatosis (~50%). This was verified in a quasi-dose dependent manner for the ACC1 protein as the rate limiting DNL enzyme. Importantly, subgroups were stratified for steatosis severity and matched for gender distribution, age and BMI to exclude main confounding factors. After overnight starvation basal αAMPK was upregulated on the mRNA and protein level, while AMPK activity was indifferent between groups. The latter was accompanied by significantly elevated fatty acid oxidation capacity in subjects with severe steatosis. We further evidenced specific lipotoxic effects of the DNL product palmitic acid/palmitate, which was significantly elevated in liver tissue and plasma of our NAFLD subjects [200]. Therefore, findings in patients primarily suffering from NAFL suggest a compensatory effect to nutrient overload, with a rise in hepatic AMPK and lipid oxidation capacity, well corresponding to the observations made in functional human in vivo studies. However, the situation likely becomes accentuated in most patients when they are advancing along the NAFLD spectrum (see Figure 1). Piro et al. studied *n* = 11 insulin resistant patients with NASH histopathology and *n* = 7 controls [201]. They found significantly reduced site specific hepatic AKT phosphorylation, corresponding to their clinical examinations. Hepatic AMPK and CPT1α transcripts were studied by RT PCR and were proven to be significantly decreased as compared to controls [201]. In accordance with these findings it was recently shown that NASH patients showed significantly reduced hepatic AMPK expression and Thr172 αAMPK phosphorylation [202]. Therefore, one hypothesis arising from available molecular evidence in humans suggests increasingly upregulated cellular DNL in early stages of NAFLD, with hepatic AMPK and fat oxidation capacity becoming initially increased in a compensatory manner. This enforces lipid oxidation as evidenced by human in vivo studies, including well defined collectives [154], to overcome this metabolic situation. However, with chronic exposure this compensation fails to sufficiently control for DNL, resulting in further triacylglycerol accumulation in the multitude of patients. Moreover, the NAFLD disease appears to be advanced by some of the adaptive mechanisms, finally resulting in impaired mitochondrial function and a rise in reactive oxygen species with insufficient detoxification potential and induction of fibrogenesis (reviewed in [203]). This corresponds to the observation of compromised hepatic AMPK activity and lipid oxidation observed in NASH patients [154,201,202]. Therefore, hepatic lipid accumulation due to insulin resistance and exaggerated DNL primarily appears as a key insult of early NAFLD, before manifestation of fibrosis or NASH (see Figure 1). Evidence to support this view comes from studies blunting ACC activity, either in transgenic rodent models, or by using pharmacologic inhibitors [138,204,205,206,207]. In several pilot trials, application of ACC inhibitors (GS-0976, NDI-010976) in human patient settings was capable of favorably influencing exaggerated DNL, hepatic steatosis, surrogate markers of fibrosis, and liver biochemistry [138,204,205,207]). The results of these studies can be seen as a proof of concept of ACC inhibition in NAFLD and are therefore of extraordinary interest from the perspective of hepatic AMPK activation approaches. However, ACC inhibition by administration the aforementioned agents over weeks produced significant hypertriglyceridemia [204,207]. This was also observed in related animal models [206] and can be explained by a rise in hepatic VLDL secretion, resulting from reduced malonyl CoA with consecutively decreased polyunsaturated fatty acid production and dysbalanced SREBP-1c/PPARα activation [208]. Future studies will have to clarify the clinical significance of these observations and potential further negative effects. Whether ACC inhibition by AMPK activation would result in comparable side effects remains elusive. However, recent studies have tested β1-specific AMPK activators in mouse and primate models showing reduced liver lipids and fibrosis through inhibition of ACC [209]. One of these agents (PXL770) is currently evaluated in phase 2b clinical trials (https://www.poxelpharma.com/en_us/product-pipeline/pxl770, accessed on 7 July 2021). Otherwise, it should be kept in mind that hepatic AMPK activity could be unimpaired or even upregulated in early NAFLD disease and effects of pharmacological stimulation could therefore depend on disease stage. We have recently identified the SLC13a5 membrane transporter (INDY, ‘I am Not Dead, Yet’) in the liver to be of relevance in human NAFLD [210]. In mIndy knockout mice reduced hepatocellular ATP/ADP ratio, activation of hepatic AMPK, induction of PGC-1α, inhibition of ACC2, and reduced SREBP-1c levels, along with attenuated DNL, have been observed [211]. SLC13a5 activity can be modulated pharmacologically and represents an interesting strategy of “upstream AMPK activation”. Furthermore, dysfunctional AMPK could have a key role in advanced NAFLD since it was recently shown that the kinase can phosphorylate the proapoptotic caspase 6 to inhibit its activation and to control for hepatocellular apoptosis in NASH [212]. Not enough phosphoproteomic studies have identified multiple new downstream targets of AMPK and the kinase is further known to favorably modify the unfolded protein response, which can also result in apoptosis. AMPK supports autophagy, keeps mitochondrial function intact, and thereby influences multiple cellular events in a beneficial manner, promising benefit for the patient along the NAFLD spectrum (see Figure 5) [57,99,111,203].

In summary, it remains to be established whether stimulation of hepatic AMPK activity will play a therapeutic role in the control of exaggerated hepatic DNL in humans. Multiple interesting functions of the kinase have been identified in recent years, exposing AMPK as a promising therapeutic option in the field of NAFLD.

## 10. Conclusions

NAFLD is a common disease comprising a wide spectrum of liver pathologies, potentially resulting in liver cirrhosis and end stage liver disease. Insulin resistance is the key driver of NAFLD pathogenesis and closely related to hepatic lipid accumulation. AMPK is a master regulator of lipid oxidation and de novo lipogenesis. In humans suffering from T2D, AMPK activation by physical activity or pharmacologic intervention can exert significant insulin sensitizing effects, mainly in skeletal muscle. Otherwise, effects of AMPK activation in human adipose tissue remain largely unknown. The role of hepatic AMPK in human fatty liver disease likely varies depending on NAFLD stage. Dysfunctional regulation of DNL and fat oxidation by AMPK is mainly observed in advanced disease stages in humans, potentially representing a promising target for pharmacological modulation. Blocking of ACC as one central downstream target of AMPK in terms of de novo lipogenesis has been proven to be principally effective and can mitigate NAFLD in humans. Yet, significant side effects were observed in some studies. Future trials will have to clarify the role of AMPK activation for mitigating exaggerated de novo lipogenesis as one major contributor of NAFLD pathophysiology.

## Data Availability

Not applicable.

## Abbreviations

ACC, acetyl CoA carboxylase; AICAR, 5-aminoimidazole-4-carboxamide-1-b-D-ribofuranoside; AMPK, AMP-activated protein kinase; ANBS, adenine nucleotide binding site; ATP, adenosine triphosphate; CaMKK2, Ca^2+^/calmodulin-dependent protein kinase kinase 2; CBM, carbohydrate binding module; CBS, cystathionine-β-synthase repeats; CD, cluster of differentiation; ChREBP carbohydrate regulatory element binding protein; CPT, carnitine palmitoyl-transferase; CVD, cardiovascular disease; DAG, diacylglycerol; DNL, de novo lipogenesis; ECM, extracellular matrix; ELOVL, elongation of very long chain fatty acid; ETC, electron transport chain; FASN, fatty acid synthase; FATP, fatty acid transport protein; FFA, free fatty acids; GLUT, glucose transporter; HOMA-IR, homeostasis model of insulin resistance; HK, hexokinase; HSC, hepatic stellate cells; LKB, liver kinase B1; L-FABP, liver fatty acid binding protein; MCD, malonyl CoA decarboxylase; MTTP, microsomal triglyceride transfer protein; MUFA, monounsaturated fatty acids; NAFLD, non-alcoholic fatty liver disease; NAFL, non-alcoholic fatty liver; NAS, NAFLD activity scoring; NASH, non-alcoholic steatohepatitis; OxPhos, oxidative phosphorylation; PDH, pyruvate dehydrogenase; PKA, proteinkinase A; PPAR, peroxisome proliferator activated receptor; PPT, protein phosphatase; PUFA, polyunsaturated fatty acids; SCAT, subcutaneous adipose tissue; SCD, stearoyl CoA desaturase; SFA, saturated fatty acids; SREBP, sterol regulatory element binding protein; TAG, triacylglycerol; TGF, transforming growth factor; Thr172, threonine 172; T2D, type 2 diabetes; VAT, visceral adipose tissue; VLDL, very low density lipoprotein.
